# A Text-Book of Operative Surgery

**Published:** 1903-12

**Authors:** 


A TEXT-BOOK OF OPERATIVE SURGERY.—Covering the surgical anatomy and operative technique involved in the operations of general surgery. Written for students and practitioners. By Warren Stone Bickham, Phar. M., M. D., assistant instructor in operative surgery, College of Physicians and Surgeons, New York; late visiting surgeon to Charity Hospital, New Orleans, etc. Handsome octavo of 984 pages, with 559 ilustrations, entirely original. Philadelphia, New York, London; W. B. Saunders & Company. 1903. Cloth, $6, net; sheep or half morocco, $7, net.

In the construction of a surgical anatomy the two most important factors, it would seem to us, are thoroughly up-to-date text matter and an accurate and precise description of the principles involved. The first of these, of course, all modern text-books worthy of the name possess, but unfortunately, the second is not so frequently accomplished. And one need not wonder at this when one realizes that in no subject in medicine do word pictures count for so little as in anatomy and surgery.

That such men as Wyeth, Senn, Halstead and Matas lent their assistance and encouragement to the above work is proof positive that the subject matter is above criticism. In the accomplishment of his second object the author has done much to elucidate matters by adhering strictly to an outline discussion of each operation, frequently comparing operations, conciseness of text, and above all, profuse illustrations. The last is by far the most pleasing, and consequently the most effective way of conveying an idea to the perplexed reader.

While realizing that this is by no means a perfect treatise on the subject, we take pleasure in recommending it as a practical one, assuring the reader that as far as our experience goes, it compares favorably with any similar work on the market. J. M. L.

